# NLC Delivery of EGFP Plasmid to TM4 Cell Nuclei for Targeted Gene Therapy

**DOI:** 10.34172/apb.2024.050

**Published:** 2024-06-22

**Authors:** Nurul Jummah, Satrialdi Satrialdi, Aluicia Anita Artarini, Anindyajati Anindyajati, Diky Mudhakir

**Affiliations:** ^1^Department of Pharmaceutics, School of Pharmacy, Institut Teknologi Bandung (ITB), Bandung 40132, Indonesia.; ^2^Department of Pharmacy, Faculty of Mathematics and Natural Science, Universitas Islam Makassar, Makassar 90245, Indonesia.; ^3^Biotechnology Laboratory, Department of Pharmaceutics, School of Pharmacy, Institut Teknologi Bandung (ITB), Bandung 40132, Indonesia.

**Keywords:** Cellular uptake, Delivery system, EGFP, NLC, Nucleic acid, Nucleus

## Abstract

**Purpose:**

This study evaluated whether a nanostructured lipid carrier (NLC) delivery system could safely and accurately deliver nucleic acids to the cell nucleus using the enhanced green fluorescent protein (EGFP)-C1 plasmid model.

**Methods:**

The NLC was formulated using the emulsification method and equipped for cationic lipid-mediated transfection with 1,2-dioleoyl-3-trimethylammonium-propane (DOTAP), which interacts electrostatically with nucleic acid. The NLC attributes, including size, polydispersity index, and zeta potential, were assessed by dynamic light scattering (DLS). The morphological structure was analyzed using transmission electron microscopy. Entrapment efficiency was evaluated by a direct method. Cellular uptake mechanisms of pEGFP-C1-NLC and the ability of pEGFP-C1 to penetrate the nucleus of TM4 cells to express EGFP were observed using confocal microscopy.

**Results:**

pEGFP-C1-NLC exhibited particle sizes in the range 56-88 nm with a particle charge range of -6.0 to+1.3 mV. The polydispersity index<0.5 showed good size uniformity, and entrapment efficiency of pEGFP-C1in the NLC was 92.06±2.295%. The NLC formulation was internalized predominantly via caveolae-mediated endocytosis, as indicated by EGFP expression following successful delivery of pEGFP by the NLC into the cells.

**Conclusion:**

NLC formulation could deliver genetic material to the nucleus and could be considered a gene therapy candidate for spermatogenesis.

## Introduction

 Sertoli cells play essential roles in regulating testicular immunity, supporting adult Leydig cells and aiding the proper function of peritubular myoid cells.​^1^ Animal model studies have shown that Sertoli cells can treat and alleviate symptoms of various conditions, including types 1 and 2 diabetes, Parkinson’s disease, Alzheimer’s disease, Huntington’s disease, Laron’s syndrome, and male infertility. This activity makes them excellent candidates for treating inflammatory conditions and as a source for cell-based therapy, as they can initiate, promote, and maintain spermatogenesis.^1-3^

 Sertoli cells are widely used in research related to cytotoxicity and spermatogenesis.^3-7^ In a study by Dr. Ralph Brinster from the University of Pennsylvania, multiple genes regulated by androgens were identified. He also showed that the expression of claudin 3 in Sertoli cells was a temporary component of the blood-testis barrier (BTB).^8^ However, his continued research was hindered by ethical clearance challenges. Previous studies suggest that CREB, Sox3, Pem (Rhox5), and DAX1 genes in Sertoli cells hold promise for developing new therapies to treat infertility.^9^ In this respect, the localization of SPATA2 protein (spermatogenesis-associated protein 2) in the adult rat testis has proven essential. Sertoli cells have also been used in gene therapy to produce SPATA2 global knockout mice with CRISPR/Cas9 technology.^8^

 In the context of gene therapy, introducing genetic materials into Sertoli cells is a complex process. Electroporation is often used to facilitate the delivery of plasmid DNA, but this method has limitations.^7^ *In vivo* studies have identified safety issues due to the adverse effects of the electroporation buffer on cell survival rates. Various methods have been developed to facilitate gene transfer into cells, such as cationic lipid-mediated transfection, direct microinjection, calcium phosphate/DNA coprecipitation, and viral vector infection. Until now, virus-mediated transfection has been the most efficient method, but it has drawbacks such as immunogenicity, complex vector construction, cytotoxicity, and limitations on the size of the inserted DNA.^2^ To address these issues, non-viral vectors need to be developed. Non-viral vectors are advantageous due to their low cost, simple use, lack of size limit on insertion, low immunogenicity, and good safety profile.^2,7,10^

 Cationic lipid materials are the most promising non-viral vectors for delivering genetic materials like nucleic acids. These lipids offer protection to nucleic acids from nuclease activity.^11^ The electrostatic interactions between the highly positively charged cationic lipids and negatively charged nucleic acids lead to complexation.^11-13^ Since the main region of the cell membrane is negatively charged, cationic lipids also help in the delivery process by promoting interaction with target cells. This interaction leads to the internalization process of the cells, mainly through endocytosis.^12,13^ Cationic lipids also facilitate the endosomal escape process through several mechanisms, which results in the efficient delivery of nucleic acids. These lipids can be incorporated into lipid nanoparticle systems such as solid lipid nanoparticles (SLNs) and nanostructured lipid carriers (NLCs). As the second generation of lipid nanoparticles, NLC offer several advantages, including higher stability, minimum drug expulsion during storage, and high encapsulation efficiency.^14,15^

 In this study, we developed a novel drug delivery system using an NLC technology that uses a cationic lipid surfactant material to introduce nucleic acids into Sertoli cells. The cationic lipid used was 1,2-dioleoyl-3-trimethylammonium-propane (DOTAP), and the pEGFP-C1 plasmid was used to develop the model methodology.^7,16-18^ This plasmid expresses enhanced green fluorescent protein (EGFP), a bright and photostable derivative of green fluorescent protein (GFP) used for tracking and labeling cells.^19^ Fluorescence-based microscopy can easily detect the expression of EGFP, making it an excellent indicator of successful delivery. We also elucidated the cellular uptake mechanism and assessed the safety of the drug delivery system in a cytotoxicity study against TM4 cells.^20^

## Materials and Methods

###  Chemicals and reagents

 The materials used for constructing the NLC, including squalene, glyceryl trimyristate, Tween 80, and Span 60, were obtained from Sigma Aldrich (St. Louis, MO, USA), while DOTAP was purchased from Avanti Polar Lipids Inc. (Alabaster, AL, USA). The pEGFP-C1 plasmid was kindly gifted by Thomas F. Meyer of the Max Planck Institute for Infection Biology (Berlin, Germany). The reagents used for *in vitro* experiments, including Dulbecco’s modified eagle medium (DMEM), fetal bovine serum (FBS), Trypsin 0.25%/EDTA, and penicillin-streptomycin were acquired from Thermo Fischer Scientific Inc. (Waltham, MA, USA). Vybrant^TM^ DiD cells-labeling solution, Hoechst 33342 (2’-[4-ethoxyphenyl]-5-[4-methyl-1-piperazinyl]-2,5’-bi-1H-benzimidazole trihydrochloride trihydrate), filipin III, amiloride, and sucrose were obtained from Sigma Aldrich (St. Louis, MO, USA). Cell Counting Kit-8 (CCK-8) was obtained from Sigma Aldrich (St. Louis, MO, USA), and Lipofectamine^TM^ 3000 Transfection Reagent was purchased from Thermo Fisher Scientific Inc (Waltham, MA, USA). TM4 cells, the mouse Sertoli cells, were received from the European Collection of Authenticated Cell Cultures (ECACC catalog number 88111401). All materials used during the experiments were of the analytical grade available.

###  NLC and pEGFP-C1-NLC formulation 

 NLC containing the pEGFP-C1 plasmid were prepared using the emulsification method with a modified literature procedure.^21,22^ In brief, the oil phase comprising 3.75% squalene (liquid lipid), 4% Span 60, 0.25% DOTAP (cationic lipid surfactant), and 0.25% glyceryl trimyristate (solid lipid), in ethanol was mixed at 65 °C with continuous stirring. In a separate vessel, 10 mM sodium citrate buffer containing 4% Tween 80 was heated to the same temperature. The two phases were mixed and homogenized using a high-shear homogenizer at 7200 rpm for 2 minutes, followed by a sonication process using a probe sonicator for 7.5 minutes.

 The pEGFP-C1 plasmid was incorporated by mixing the plasmid solution into the NLC, followed by incubation at 4 °C for 30 minutes. pEGFP-C1-NLC was stored at 4 °C for subsequent experiments.

###  NLC particle size, polydispersity index, and zeta potential characterization 

 The physical properties of pEGFP-C1-NLC were determined by analyzing their hydrodynamic diameter, particle size distribution (PDI), and zeta potential. The dynamic light scattering (DLS) method was used to determine the particle size and distribution with a Delsa^TM^ Nano C Particle Size Analyzer (Beckmann Coulter, Brea, CA, USA). The zeta potential was estimated using the laser Doppler electrophoresis method with the same instrument.

###  NLC microstructure analysis with TEM

 The blank NLC and pEGFP-C1-NLC microstructure was analyzed by transmission electron microscopy (TEM) (JEOL 1010, Peabody, MA, USA). In brief, 10 µL of the sample was dispensed onto the specimen. Then, a 400-mesh grid tool was placed on the specimen bearing the nanoparticle droplets and was left for 1 minute. Next, the droplets were discarded, and 10 µL of phosphotungstic acid (PTA) was applied to the grid for 1 minute. After discarding the remaining droplets, the specimen was allowed to dry for 15 minutes. Finally, the dry sample on the grid was analyzed by TEM (80.0 KV, 30,000x magnification).

###  NLC entrapment efficiency measurement

 Entrapment efficiency (EE) of the pEGFP-C1 plasmid in the NLC system was determined by a direct method using a modified literature procedure.^23^ Firstly, the separation of pEGFP-C1-NLC from non-encapsulated pEGFP-C1 plasmid was carried out by centrifugation (MWCO 5 kDa) at 16 000 rpm for 45 minutes using a HERMLE Z36 HK centrifuge (Germany). The encapsulated pEGFP-C1 plasmid was analyzed using a microplate reader (Thermo Scientific Multiskan Go, Waltham, MA, USA) at a wavelength of 260 nm. EE was calculated using the following equation:


$$EE\left(\%\right)=\frac{W\left(pEGFP-C1\;in\;NLC\right)}{W\left(Initial\;pEGFP-C1\right)}\times 100$$

###  Stability of NLC

 The stability of pEGFP-C1-NLC complex stored at 4 ºC was determined after 2 and 7 days by measuring particle size, PDI, and zeta potential.^24^

###  TM4 cell culture

 TM4 cells were cultured in DMEM supplemented with 10% FBS and penicillin-streptomycin, and were incubated at 37 °C under 5% CO_2_/air and passaged at 90% confluency.

###  Safety assessment of NLC in TM4 cells

 The safety of NLC was evaluated *in vitro* by testing its effect on the viability of TM4 cells. TM4 cells were seeded on a 96-well plate at a density of 5000 cells per well. After 24 hours of incubation, the cells were treated with fresh medium containing different concentrations of NLC and then incubated for another 24 hours. To determine cell viability, the CCK-8 assay was used, and the absorbance of the reagent was measured at 450 nm.^25^ Untreated cells were used as a reference for 100% cell viability. At least three independent experiments were conducted.

###  Cellular uptake efficiency of NLC into TM4 cells

 The cellular uptake mechanism of pEGFP-C1-NLC was qualitatively elucidated using confocal laser scanning microscopy (CLSM). To track the nanoparticles within cells, NLC was modified with a fluorescence probe called DiD (Ex/Em = 644/665 nm) to investigate the effect on ATP of the cellular uptake process. TM4 cells were seeded on a confocal dish at a density of 1 × 10^5^ cells/dish for 24 hours. Next, the TM4 cells were transfected with DiD-labeled NLC for 1 hour, and then the nuclei of the cells were stained using Hoechst 33342 for 15 minutes. CLSM observation was conducted using an Olympus FV1200 (Tokyo, Japan). The cellular uptake efficiency was compared for cells incubated at 4 °C and 37 °C, representing the ATP-independent and ATP-dependent cellular uptake processes, respectively.^26^ To calculate the cellular uptake efficiency, the intensity of DiD (Ex/Em = 644/665) was measured semi-quantitatively using ImageJ software.^27^

###  Elucidation of endocytosis pathway of NLC in TM4 cells

 To better understand the endocytosis pathway, cellular uptake mechanisms were elucidated qualitatively by CLSM using specific endocytosis pathway inhibitors. The inhibitors used were 0.4 M sucrose for clathrin, 5 µg/mL filipin III for caveolae, and 0.1 mM amiloride for macropinocytosis pathways.^28^ TM4 cells were seeded at a density of 1 × 10^5^ cells/dish and incubated for 24 hours. The cells were transfected with the medium containing the specific inhibitor for 30 minutes, and were treated with DiD-labeled NLC for 1 hour. The cells were washed and stained with Hoechst 33342 for 15 minutes before CLSM observation. ImageJ software was used to calculate cellular uptake efficiency semi-quantitatively by measuring DiD intensity.

###  Analysis of EGFP expression

 The delivery efficiency of the pEGFP-C1 plasmid was evaluated by examining the expression of GFP qualitatively using CLSM.^20^ Firstly, TM4 cells were seeded onto a confocal dish at a concentration of 1 × 10^5^ cells/dish and incubated for 24 hours for cell attachment. Then, the cells were transfected with pEGFP-C1-NLC and incubated for 1 hour to facilitate the uptake of the plasmid. After replacing the growth medium, the cells were incubated for an additional 23 hours. To observe the cells using CLSM, Hoechst 33342 was added to stain the TM4 cell nuclei. The intensity of GFP was analyzed semi-quantitatively using ImageJ software. As a positive control, Lipofectamine 3000 was used to enhance the transfection with the pEGFP-C1 plasmid.

###  Statistical analysis

 Data analysis was carried out using theoretical and statistical methods. Measurement results were presented as mean ± standard deviation. Statistical analysis was performed by unpaired t-student analysis using Minitab 20. Results were considered statistically significant when *P < 0.05, **P < 0.01, and *** *P* < 0.001.

## Results and Discussion

###  NLC formulation and characterization

 In the present study, we developed an NLC drug delivery system for the pEGFP-C1 plasmid. The NLC system has demonstrated a greater ability to retain drug compounds at higher concentrations in water, and allows for the creation of controlled pharmaceutical dosage forms, improves drug chemical stability, and can be produced on a large scale.^29^ Design of the delivery mechanism was based on regulating biophysical interactions.^21^ We employed a non-ionic surfactant system consisting of Tween 80 and Span 60 to maintain colloidal stability. The use of non-ionic surfactants with relatively low toxicity can reduce the negative impact on the cells. We also added DOTAP, a cationic lipid surfactant with a positively charged symmetrical Gemini structure that can bind with negatively charged pEGFP-C1 through electrostatic interactions.^30-32^ The presence of DOTAP can also drive the efficient delivery of nucleic acids by facilitating cellular uptake and endosomal escape processes.^33^

 pEGFP-C1-NLC complex was constructed using the simple and reliable emulsification method. Lipid and water phases were combined at 65 °C using a high-shear homogenizer to form the emulsion system, followed by a sonication process to break particles into NLCs. The pEGFP-C1 plasmid was added, followed by 30 minutes of incubation to facilitate the electrostatic interaction and incorporation into the positively charged NLC. The resulting pEGFP-C1-NLC particles were characterized by size, size distribution, and zeta potential.^21,22^

 Both pEGFP-C1-NLC formula and preparation process were optimized. In the first stage of formula optimization, our objective was to determine a surfactant/oil (S:O) ratio suitable to produce 50-100 nm particles with PDI < 0.50, a size distribution considered optimal for internalizing into TM4 cells. Tween 80 and Span 60 were used as the base non-ionic surfactants, cationic surfactant DOTAP was added to package negatively charged pEGFP-C1 by electrostatic interaction, while squalene (liquid lipid) and glyceryl trimyristate (solid lipid) were used as oils.

 In a preliminary formula, an S:O ratio of 1.85 resulted in a particle size of 51.90 ± 5.75 nm and PDI 0.29 ± 0.13. Addition of DOTAP resulted in S:O 1.91, particle size 127.20 ± 5.95 nm, and PDI 0.31 ± 0.02. Since the particle size was not within the required range, we performed process optimization, varying temperature (50 and 65 °C), stirring rate (5000, 6000, and 7200 rpm), and sonication time (2, 5, and 7.5 minutes). Finally, at 65 °C, 7200 rpm, and 7.5 minutes sonication, an S:O ratio of 2.13 gave particle size 90.00 ± 7.18 nm, and PDI 0.306 ± 0.025 (Table 1). The NLC composition corresponding to S:O 2.13 comprised 4% Tween 80, 4% Span 60, 0.25% DOTAP, 3.75% squalene, and 0.25% glyceryl trimyristate. Subsequent incorporation of pEGFP-C1 provided pEGFP-C1-NLC with particle size 87.60 ± 6.15 nm, and PDI 0.297 ± 0.006 (Table 1).

**Table 1:** Characterization of blank-NLC and pEGFP-C1-NLC.

**Formulation**	**Particle size (nm)**	**Polydispersity index**	**Zeta potential (mV)**	**Entrapment efficiency (%)**
Blank-NLC	90.00 ± 7.18	0.306 ± 0.025	+ 4.24	n.a.
pEGFP-C1-NLC	87.60 ± 6.15	0.297 ± 0.006	+ 1.29	92.06 ± 2.295

Note: Results obtained from n = 3 experiments.

 The particle size and size distribution were analyzed by DLS. As shown in Table 1, both blank NLC and pEGFP-C1-NLC displayed a comparable particle size less than 100 nm. A particle size of less than 200 nm can facilitate penetration into the cell membrane and the nucleus, which is required for delivering nucleic acids such as plasmid DNA.^34,35^ Moreover, the resulting particle showed a homogenous distribution, indicated by PDI < 0.5. Additionally, the preparation method demonstrated high reliability, as revealed by a low standard deviation for particle size and PDI.

 Zeta potential of the NLC was measured with a laser Doppler electrophoresis method. The zeta potential reflects how effectively nanoparticles interact with biological systems. Materials with a neutral or negative surface charge are more likely to circulate for longer periods and accumulate less in the liver and spleen.^36^ In the presence of the pEGFP-C1 plasmid, the zeta potential for the NLC decreased (Table 1), indicating the successful formation of a complex through electrostatic interactions. This conclusion was supported by the entrapment efficiency, which revealed that over 90% of the pEGFP-C1 plasmid had successfully bonded with the NLC system.

**Figure 1:**
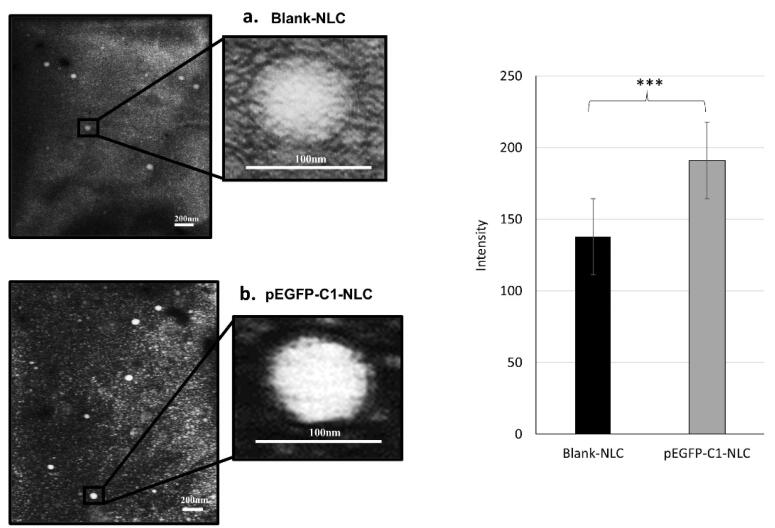
Morphologies of (a) blank NLC and (b) pEGFP-C1-NLC characterized by TEM

 The microstructure of the NLC was analyzed using TEM, employing a negative staining method with PTA, resulting in a dark background with a brighter image of the nanoparticles. According to the TEM images (Figure 1), blank NLC and pEGFP-C1-NLC displayed a spherical shape with a diameter ranging from 60 to 100 nm. This result confirmed the particle size determined by the DLS method. Addition of pEGFP-C1 plasmid increased the rigidity and compactness of the particles compared to the blank NLC. This is attributed to electrostatic interactions and the formation of a more rigid complex between plasmid DNA and the NLC. Hence, the interaction between pEGFP-C1 plasmid and the NLC system was stable and caused no change to the shape and size of the particles. Moreover, the negative shift in zeta potential indicates incorporation of anionic pEGFP-C1 plasmid into the NLC.

 TEM images of pEGFP-C1-NLC were of higher intensity than those of blank-NLC (Figure 1). This difference in intensity is attributed to the pEGFP-C1, which provides additional electron-dense material that scatters more electrons, resulting in brighter areas under TEM. Additionally, the heavy metal PTA staining used in TEM microscopy may interact more with the phosphate groups in the pEGFP-C1-loaded NLC, thus increasing contrast. In summary, the enhanced intensity results from the differences in composition between the two samples, which leads to increased electron scattering. These results suggest that the pEGFP-C1 has been successfully loaded.

###  Stability of NLC formulation

**Figure 2:**
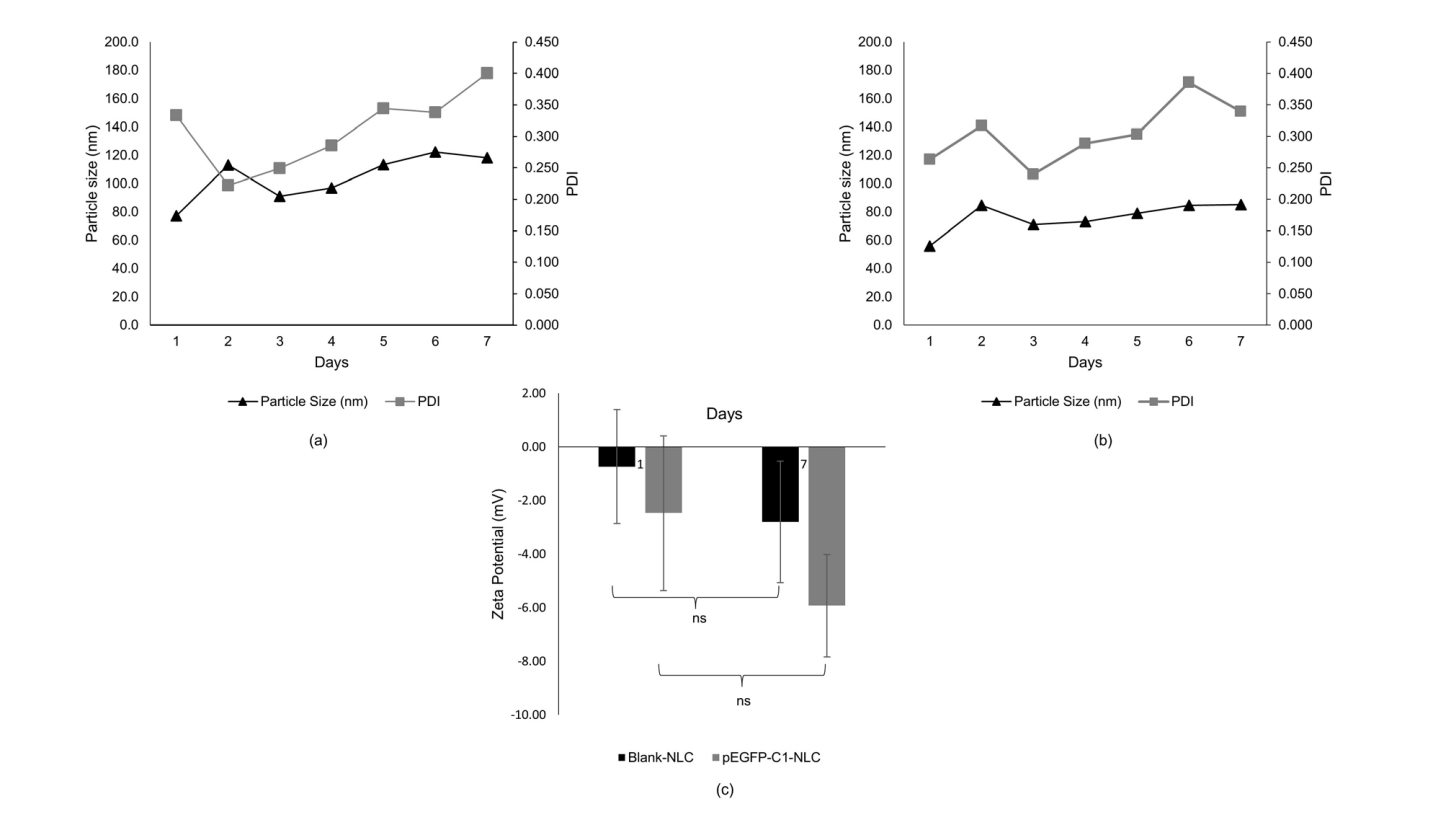
Short-term stability of (a) blank-NLC and (b) pEGFP-C1-NLC at 4 °C for 7 days; and (c) zeta potentials for blank NLC and pEGFP-C1-NLC on days 1 and 7 (n = 3)

 To evaluate the stability of the developed nanoparticles, blank NLC and pEGFP-C1-NLC were stored at 4 °C for 7 days and the particle size and PDI were measured over the period.^24^ By observation, both blank-NLC and pEGFP-C1-NLC maintained the same appearance without precipitation after 7 days. For both NLCs, particle size in range 50-150 nm and PDI < 0.5 were also stable (Figure 2 and Table S1). The high stability of blank-NLC and pEGFP-C1-NLC was also evidenced by the non-significant decrease in zeta potential between days one and seven (Table S2).

###  Toxicity of NLC in TM4 cells

**Figure 3:**
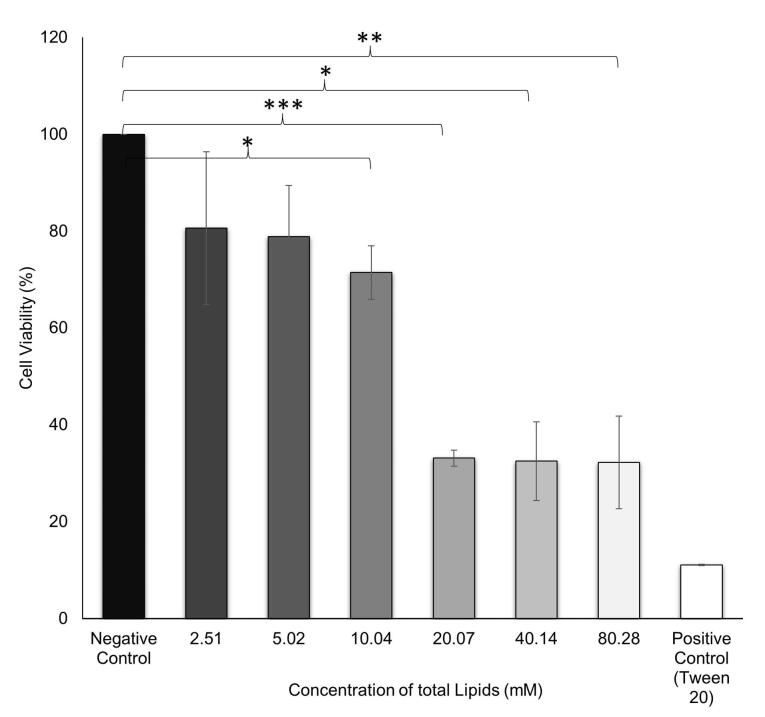
Cell viability test by CCK-8 assay in TM4 cells treated with various concentrations of total lipids for 24 h. Data are representative of three individual replicates. *P< 0.05, **P< 0.01, ***P< 0.001 in comparison to the untreated group (negative control)

 The toxicity of the NLC system was assessed with a CCK-8 cell viability assay. The result showed an IC_50_ value of 19.16 ± 7.33 mM of total lipids. Total lipids in the range 2.51-10.04 mM showed cell viability > 70%, which indicated a safe concentration (Figure 3). This concentration was then used as a reference for further *in vitro* testing. A previous study also conducted cell viability studies as evidence of the biocompatibility of lipid nanoparticles at concentrations < 0.80 mM total lipids in *in vitro* tests.^37^

###  Cellular uptake efficiency of NLC into TM4 cells

**Figure 4:**
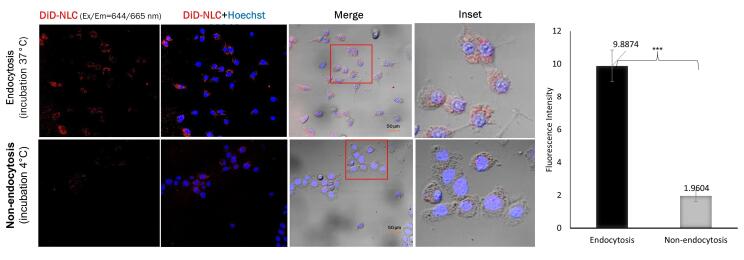
Cellular uptake studies in TM4 cells. Cells were incubated for 1 h with DiD-labeled NLC (red channel). Cell nuclei were counterstained with Hoechst 33342 (blue channel). Insets show the boxed areas at a higher magnification

 Nanoparticles are usually taken up by cells through endocytosis. To monitor the uptake of our NLC system into TM4 cells, DiD labeling was used. The amount of DiD-labeled NLC taken up by the cells through endocytosis was five times higher than that by the non-endocytosis pathway, and this difference was statistically significant with *P* < 0.001 (Figure 4).

**Figure 5:**
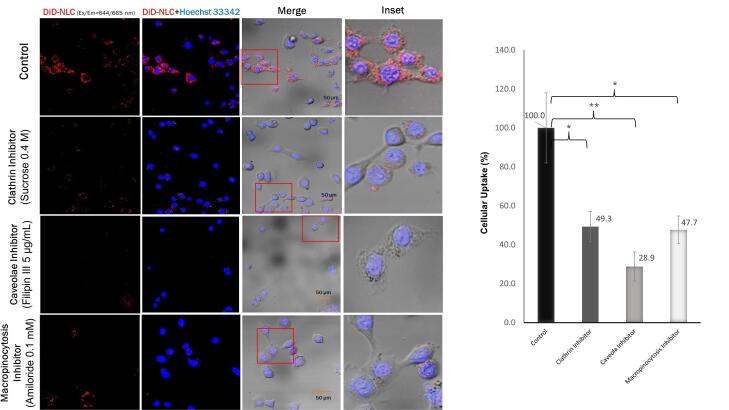
Uptake of NLC in the presence of different specific inhibitors in TM4 cells. Cells were incubated for 1 h with DiD-labeled NLC (red channel). Cell nuclei were counterstained with Hoechst 33342 (blue channel). Insets show the boxed areas at a higher magnification

 The endocytosis pathway, whether via caveolae, macropinocytosis, or clathrin, was also investigated using specific pathway inhibitors. The results in Figure 5 indicate that endocytosis of NLC through caveolae was inhibited by 70% compared to the control. It has been reported that 50-100 nm nanoparticles pass into cell targets predominantly via caveolae, followed by macropinocytosis and clathrin.^38-40^ Caveolar endocytosis comprises the formation of spherical 50-60 nm caveolae driven by an integral membrane protein called caveolin and a peripheral membrane protein known as cavin. In addition to the favorable size of the approximately 80 nm nanoparticles, the caveolar pathway was also triggered by the binding of ligands to receptors concentrated in the caveolae regulated by kinases and phosphatases. It is likely that the kinase enzyme pathway caused lipid phosphorylation of the NLC components, facilitating its entry into the phospholipid-containing cell membrane.^41^

###  Analysis of EGFP expression

 The uptake of nanoparticles into cells is influenced by endosomal escape, which is known to be the rate-limiting barrier for such systems. Bioactive molecules are often trapped in endosomal vesicles and degraded in lysosomal compartments, which requires effective strategies to facilitate endosomal escape and increase the bioavailability of the delivered bioactive agent. Novel methods for the intracellular delivery of bioactives are needed to overcome this barrier. These strategies include membrane fusion, which is possible in NLC delivery systems with synthetic cationic lipids, namely DOTAP.^33^

**Figure 6:**
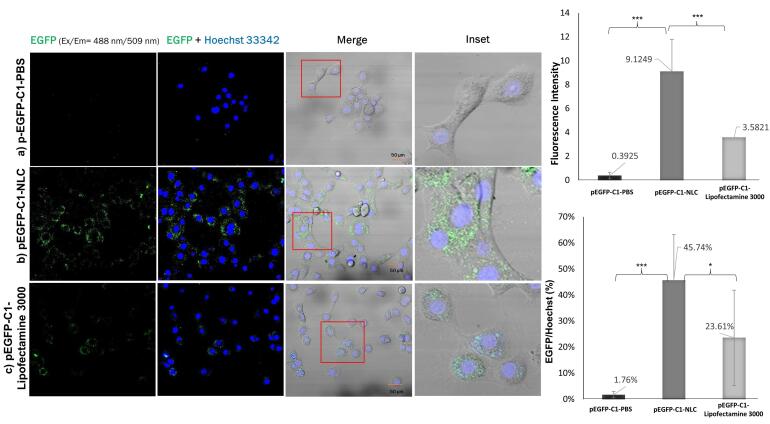
EGFP expression in TM4 cells. Cells were incubated for 24 h with the different delivery systems a) pEGFP-C1-PBS 50 ng, b) pEGFP-C1-NLC 50 ng, and c) pEGFP-C1-Lipofectamine 3000 50 ng. Cell nuclei were counterstained with Hoechst 33342 (blue channel). Insets show the boxed areas at a higher magnification

 The pEGFP-C1-NLC formula was able to enter the nucleus and express EGFP due to the delivery of the pEGFP-C1 plasmid by NLC, as shown in Figure 6. In comparison, pEGFP-C1 dispersed in PBS showed no expression of EGFP because PBS could not internalize pEGFP-C1 into the cells. The results also showed that EGFP expression with pEGFP-C1-Lipofectamine 3000 was approximately half that with pEGFP-C1-NLC. The transfection efficiency data based on the EGFP:Hoechst ratio showed that EGFP expression with pEGFP-C1-NLC was twice that with pEGFP-C1-Lipofectamine 3000.

 In a study by Buck et al in 2020, the efficiency of DOTAP and Lipofectamine 3000 as carrier agents was also examined. The results showed that DOTAP/cholesterol was only 30% as efficient as Lipofectamine 3000 in the delivery of pEGFP.^42^ In contrast, our study showed a higher transfection efficiency using DOTAP (45.7%) than lipofectamine 3000 (23.6%) after 24 hours, demonstrating the increased efficiency of DOTAP in binding pEGFP in our NLC formula. Another study by Han et al in 2014 examined transfection efficiency of A549 lung cancer cells using an NLC for drug delivery with a transferrin (Tf)-modified ligand.^43^ The NLC/pEGFP and Tf-NLC/pEGFP transfection efficiencies were approximately 25% and 33% after 24 hours, respectively. Our results were better than these, likely due to the 92% entrapment efficiency (EE) of pDNA by the DOTAP-modified NLC formula, compared to 83% in Han and colleagues’ study. In addition, Zhang et al reported approximately 89% EE of hyaluronic acid-pDNA in an NLC, and 39% transfection efficiency in A549 lung cancer cells after 36 hours.^44^ Therefore, the high EE of pDNA in pEGFP-C1-NLC is essential for the higher transfection efficiency of our system.

 Using an NLC with ligand surface modification, Klein et al in 2022 reported that LNP smaller than 100 nm were easily localized in the testes.^45^ Therefore, our 56-87 nm pEGFP-C1-NLC are expected to effectively accumulate in the testes. Once the NLC attaches to the surface of TM4 cells, it enters the cells and releases its cargo. We demonstrated that the pEGFP-C1-NLC particles were internalized into TM4 cells predominantly by caveolae-mediated endocytosis (41%), followed by the macropinocytosis (30%), and clathrin (29%) pathways. Interestingly, although the pEGFP-C1-NLC is small, the particles also enter the cells via macropinocytosis, a pathway usually associated with a size range of 0.2-5 μm.^46^ Similar induction of actin rearrangement and membrane ruffling is likely to occur by the pEGFP-C1-NLC, as reported previously.^47-49^ The entry of pEGFP-C1-NLC by the caveolar pathway avoids lysosomal uptake, thereby avoiding lysosomal degradation, allowing pEGFP-C1 transport to the nucleus. The caveolar pathway is known to directly reach the endoplasmic reticulum (ER), as used in SV40 internalization, by moving along the microtubules.^50^ In the case of pEGFP-C1-NLC, transporting pDNA to the ER lumen presumably increases the delivery efficiency to the nucleus via the ER-nucleus route. Therefore, our NLC delivery system with small particles (50-100 nm) without ligand surface modification showed high pEGFP-C1 transfection efficiency to the nucleus due to the specific entrance pathways of pEGFP-C1-NLC. This delivery system could provide a novel solution to targeted therapy, particularly in male infertility, and this study has also provided solutions for addressing the challenge of the blood-testis barrier associated with Sertoli cells.

## Conclusion

 Critical for successful delivery of pDNA to the cell nucleus to encode EGFP is the NLC uptake mechanism. This study identified the caveolar endocytosis pathway to be the predominant uptake mechanism, and transport to the ER lumen via the ER-nucleus route. The cationic surfactant DOTAP-containing NLC is a promising candidate for safe, effective nuclear targeted gene therapy and this study has demonstrated its potential for diseases associated with Sertoli/TM4 cells and in treating male infertility.

## Acknowledgments

 The authors thank to ITB-Olympus Bioimaging Center (IOBC), Evident Scientific and PT. Wadya Prima Mulia for providing access of instrument and support.

## Competing Interests

 The authors declare that they have no conflict of interest.

## Ethical Approval

 Not applicable.

## Supplementary Files

Supplementary file 1 contains Tables S1 and S2.
