# Supplementary material for: NLC Delivery of EGFP Plasmid to TM4 Cell Nuclei for Targeted Gene Therapy
Source: Adv Pharm Bull. 2024 Jun 22;14(3):613–22. doi: 10.34172/apb.2024.050 (PMC11530889; doi:10.34172/apb.2024.050)
Supplement: Supplementary file 1 — contains Tables S1 and S2. [file apb-14-613-s001.pdf]

Supplementary Data
Doi: 10.34172/apb.2024.050

NLC Delivery of EGFP Plasmid to TM4 Cell Nuclei for Targeted Gene Therapy

Nurul Jummah[1,2], Satrialdi Satrialdi[1], Aluicia Anita Artarini[3], Anindyajati Anindyajati[3], Diky Mudhakir[1]*

[1]Department of Pharmaceutics, School of Pharmacy, Institut Teknologi Bandung (ITB), Bandung 40132, Indonesia.
[2]Department of Pharmacy, Faculty of Mathematics and Natural Science, Universitas Islam Makassar, Makassar 90245, Indonesia.
[3]Biotechnology Laboratory, Department of Pharmaceutics, School of Pharmacy, Institut Teknologi Bandung (ITB), Bandung 40132, Indonesia.

***Corresponding author details**
Diky Mudhakir, Phone: +62-22-2504852, Email: mudhakir@itb.ac.id

Supplementary Data

Table S1. Short-term stability of blank-NLC and pEGFP-C1-NLC at 4°C for 7 days.

Formulation	Days	Particle Size (nm)	Polydispersity Index
Blank-NLC	1	77.2 ± 7.9	0.333 ± 0.077
	2	113.1 ± 21.6	0.222 ± 0.145
	3	91.2 ± 6.6	0.249 ± 0.022
	4	97.0 ± 4.9	0.286 ± 0.011
	5	113.3 ± 11.1	0.344 ± 0.037
	6	122.2 ± 7.4	0.338 ± 0.049
	7	118.1 ± 13.1	0.400 ± 0.065
pEGFP-C1-NLC	1	56.0 ± 2.1	0.263 ± 0.032
	2	84.6 ± 7.4	0.317 ± 0.043
	3	71.1 ± 10.4	0.240 ± 0.113
	4	73.1 ± 2.2	0.289 ± 0.018
	5	79.0 ± 7.3	0.303 ± 0.013
	6	84.5 ± 4.2	0.386 ± 0.029
	7	85.2 ± 1.8	0.340 ± 0.034

Note: Results obtained from n=3 experiments.

Table S2. Zeta potentials for blank-NLC and pEGFP-C1-NLC on the days 1 and 7.

Formulation	Days	Zeta Potential (mV)
Blank-NLC	1	-0.74 ± 2.13
	7	-2.81 ± 2.27
pEGFP-C1-NLC	1	-2.47 ± 2.88
	7	-5.93 ± 1.91

Note: Results obtained from n=3 experiments.

supplementary
